# Managing information gaps on caregivers of psychotic patients in primary health settings of Yogyakarta, Indonesia

**DOI:** 10.15171/hpp.2018.21

**Published:** 2018-04-18

**Authors:** Carla R. Marchira, Warih A. Puspitosari, Ida Rochmawati, Siti Mulyani, Irwan Supriyanto

**Affiliations:** ^1^Department of Psychiatry, Faculty of Medicine, Gadjah Mada University, Yogyakarta, Indonesia; ^2^Department of Psychiatry, Faculty of Medicine and Health Sciences, Muhammadiyah University, Yogyakarta, Indonesia; ^3^Wonosari district hospital, Gunung Kidul, Yogyakarta, Indonesia; ^4^Kasihan 2 Primary health center, Bantul, Yogyakarta, Indonesia

**Keywords:** Brief psychoeducation, Family caregivers, Psychotic disorders, Primary health center

## Abstract

**Background:** Information gaps hinder the management of psychotic patients. Incorporating mental health services into primary care might solve the problem. Health workers can be trained to provide psycho-education for caregivers of psychotic patients.

**Methods:** This study was a two stage experimental study. In the first stage, 43 health workers were trained to provide a psycho-education for caregivers of psychotic patients. Next, 10 health workers were selected to provide the psycho-education. Participants were family caregivers of psychotic patients (n = 113) randomly assigned to control and intervention groups. Pre- and post-tests were conducted to assess the results. Statistical analyses were conducted using paired t tests and analysis of covariance (ANCOVA).

**Results:** The intervention group scored higher for Knowledge of Psychosis at post-test. Both groups showed significant increase of knowledge (paired t tests: P<0.001; P<0.001; respectively) and the increases were not significantly different between the groups (ANCOVA: P=0.057).

**Conclusion:** Psycho-education was applicable in primary care settings. Training primary care health workers is a feasible method to address information gaps in the management of mental health problems.

## Introduction

Lack of proper information about psychotic disorders increases difficulties for caregivers to manage the disorders. Most caregivers experience uncertainties about the nature of the disorder, medical treatment options, and the prognosis of the disorder.^1,2^ Cultural interpretations of the disease often lead the caregivers to seek for non-medical treatment and delay proper medical treatment.^3^

Medical information about the nature of psychotic disorders and treatment influence how families provide care. Psycho-educational interventions are aimed at providing families and persons living with the disorders accurate information that can serve to improve management of care, lower relapse rate, increase adherence to treatment, and lower the stigma.^4-6^

Lack of mental health facilities has lead Indonesian to rely on primary health care for mental health services. Unfortunately, most of the health workers are not trained to provide psycho-education for mental disorders, particularly psychotic disorders. In this study, we applied a brief psycho-education module on the knowledge of psychosis we have developed^7^ in primary health care settings in Yogyakarta, Indonesia.

## Materials and Methods

### Setting

Yogyakarta is a province of Indonesia with a population of 3.6 million people in 2016 and is the second in the number of patients with schizophrenia nationally. This population is served by 25 psychiatrists who practice primarily in 1 state mental hospital, 5 district hospitals and several private hospitals with psychiatric units, a university teaching hospital and a private psychiatric hospital. Yogyakarta is also a centre of Javanese culture, and most families resort to diverse Javanese and Islamic healers in response to severe mental illnesses. Literacy about medical care for mental illnesses is quite low, emphasizing the importance of providing accurate information to those with an illness and family caregivers.

### Study design and participants

This study was a two stage experimental study. For the first stage, primary health center workers were trained to conduct brief psycho-education on psychosis to caregivers of patients with psychotic disorders. The subjects for the training were 43 health workers in three primary health centers in Yogyakarta (12 from Kasihan 2, 12 from Dlingo 1, and 19 from Wonosari 3). The primary health centers were selected based on the availability of mental health programs. The participants were doctors, nurses, and midwives responsible for mental health service in each center. The training was conducted in 4 sessions, each session lasting 1-2 hours. Subjects were trained on the content and how to use the brief interactive psycho-education module on psychosis that we had developed. Pre- and post-test was conducted using the Knowledge of Psychosis instrument we developed and 10 health workers from each primary health center were selected as educators for the next step of the study.

For the second step, the selected health workers were asked to provide a brief psycho-education on psychosis using the same module they used for training. The subjects for the brief psycho-education were family caregivers of patients with psychotic disorders based according to International Statistical Classification of Diseases and Related Health Problems-10th Revision (ICD-10). Family caregivers of patients with affective disorders with psychotic features and those who did not complete psycho-education were excluded. The caregivers were people living under the same roof with the patient, responsible for the treatment and care of the patient, and spent at least 35 hours per week in contact with the patient.^8^ If more than one caregiver was identified, the main caregiver was the one responsible for the patient’s medication and/or who had the most frequent contact with the patient.

There were 113 subjects who consented to participate from the three public health centers. The subjects were than randomly assigned to the control and intervention groups.

The interventions were conducted in four sessions, once per week for 1 month. The education was conducted individually for each caregiver. Sessions were interactive and lasted 1-2 hours. The four sessions focused on: (*a*) the definition and aetiology of psychotic disorders; (*b*) signs and symptoms of the disorders; (*c*) treatment and care for the disorders; and (*d*) support system, services available, and signs and symptoms of exacerbation or relapse.

The standard care received by the control subjects were standard family education. The education was comprised of a brief explanation on the diagnosis, treatment regiment, and prognosis of the disorder. Unlike our module, the education was unstructured. The standard education was also provided by primary care health workers and on average lasted only for fifteen minutes.

### Module and instrument

The modules used in this study were adapted for Javanese culture. The terms used in the modules were matched with words and phrases often used by caregivers to express the psychotic symptoms the patient experienced. The terms reflect classic Javanese cultural ideas about psychotic illness as well as religious ideas associated with understanding illness and seeking care in Javanese society. The educators were trained to provide explanation using terms in accordance to the Javanese culture and understanding of mental illnesses. The modules were designed using lay terms easily understandable in the local context. Each module provided the persons conducting the intervention the purpose of the session, duration, delivery method, knowledge content, and opening and closing remarks.^7^

Assessment instruments used in this study were the KOP adopted from Compton et al^9^ and used to assess the knowledge of the caregivers concerning psychotic disorders. KOP is a brief, self-administered knowledge test, comprised of 18 multiple-choice questions. These questions assess the caregiver’s knowledge on the aetiology (from biological to psychosocial perspectives), signs and symptoms (derived from diagnostic criteria), treatment (drugs and psychosocial treatment), appropriate care providers (from whom they should seek for treatment), information inquiry (reliable sources of information), and support system required for psychosis. The caregivers were scored for the number of correct answers. The KOP was used to assess the knowledge of family caregivers before the control and intervention training with a pre-test and one month after the brief psycho-education with a post-test.

### Data analysis

The data was statistically analyzed using SPSS version 17. The pre- and post-test scores for KOP were compared using paired *t* test to find if there were significant differences in the pre and post-test scores. Analysis of covariance (ANCOVA) using pre-test score as covariate with 95% CI was conducted to assess for the score increase/decrease between the groups. Statistical significance was defined as *P* < 0.05.

## Results

Demographic characteristics of the family caregivers are presented in Table 1. Statistical analyses showed that pre-test scores were not significantly different between control and intervention groups but post-test scores showed that the intervention group scored significantly higher (Table 2). Both groups showed significant increase in post-test scores (paired *t* tests: *P* < 0.001; *P* < 0.001; respectively), but the intervention group showed greater increase. Nevertheless, analyses using ANCOVA with pre-test as covariate to adjust for the post test scores showed that the increases were not statistically different between the groups (ANCOVA; *P* = 0.057) (Table 3).

## Discussion

In this study, we demonstrated that providing a brief psycho-education in Yogyakarta using an education module adapted for Javanese culture is feasible and effective, and that this intervention significantly increased knowledge about psychotic illness in the intervention group compared to the control group in primary health care settings.

Lack of medical knowledge concerning psychotic disorders and treatment efficacy is known to increase non-adherence with treatment, resulting in relapses and rehospitalisation.^10^ Relapses increase the disease burden and worsen stigma. Psycho-education is known to effectively increase caregiver knowledge concerning psychotic disorders, even when delivered by primary care workers as shown in this study. Despite this fact, it is not routinely used in health services in low resource settings such as Yogyakarta.

In this study, both the control and intervention groups showed an increased score of KOP. Caregivers in both control and intervention groups are likely to receive information about the disorders from other sources. The questions in the pre-test might stimulate them to seek for answers for knowledge they are lacking from other sources. Since the instrument used in the pre- and post-test was the same, and both groups’ scores improved, the use of the similar instrument for pre- and post-test might result in an inevitable bias. We cannot evaluate the extent to which increased knowledge of caregivers was due to information from sources other than the psycho-education intervention. Nevertheless, the knowledge gained by the control groups most likely is culturally associated knowledge that would likely render proper medical treatment.^3^ Another possible cause for similar results in both groups was contamination from intervention to control group. Due to frequent contacts, sharing of experiences and knowledge are common practice among caregivers of psychotic patients, particularly information that they found useful.

Untreated or poorly treated psychotic disorders have profound social and medical consequences. Therefore, proper and timely management of the early phases of psychotic disorders are important as preventive measures as well as a firm foundation for future management. Ineffective management may result in discontinuing treatment, relapse of symptoms, increase in the risk of treatment resistance and higher stigma.^1,11^

In Indonesia, low mental health resources, lack of knowledge and stigmatization result in delayed treatment. Local understandings of illness and lack of medical information about the nature of psychotic illness and treatment influence how families provide care. These circumstances may be a significant source for non-adherence, in some cases leading to early termination of treatment.^12^ It has been reported that 67% of caregivers of patients with psychotic disorders sought help from traditional healers prior to their seeking medical help.^3^

For future directions, our ongoing project is to explore the impacts of increased knowledge on the management of the disorder by caregivers after brief psycho-education by primary health care workers, particularly on relapse prevention, contact with mental health service providers, and adherence to pharmacotherapy.^13^ In our previous study in a hospital based setting, the brief psycho-education reduced rehospitalisation (6% in the intervention group compared to 18% in the control group).^7^ It has been reported that in Indonesia, within two years patients with schizophrenia relapse 1.48 times on average with the highest recurrence frequency within two years is four times.^14^

In conclusion, brief psycho-education on psychosis for caregivers of patients with psychosis by health workers in primary health care is possible and proved to be effective. This type of training might be used to address information gaps among caregivers of patients with mental health problems in the primary care settings, particularly in primary care settings in Indonesia which are lacking sufficient mental health resources. Therefore, this approach can serve to improve the management of psychotic patients, increase adherence to treatment, and reduce relapses.

## Ethical approval

Informed consent was obtained from all subjects. The protocol of this study was approved by the Ethical Committee of the Faculty of Medicine, Gadjah Mada University, Yogyakarta.

## Competing interests

The authors declare that they have no competing interests.

## Authors’ contributions

CRM developed the study design and was responsible for data acquisition; WAP and IR collected and extracted data; SM was responsible for the psycho-education training; Irwan Supriyanto conducted analysis and drafted the manuscript

## Acknowledgments

We would like to thank the staffs at Kasihan 2, Dlingo 1, and Wonosari 3 primary health center who were involved in the psycho-education.

**Table 1:** Demographic characteristics of subjects (n = 113)

**Variables**	**Groups**			
	**Intervention**	**%**	**Control**	**%**
Sex				
Male	26	46.43	30	52.63
Female	30	53.57	27	47.37
Education				
Elementary school	22	39.29	20	35.09
Junior high school	6	10.71	10	17.54
Senior high school	16	28.57	14	24.56
College graduate	12	21.43	7	12.28
Occupation				
Farmer	11	19.64	10	17.54
Labor	10	17.86	9	15.79
Merchant	3	5.36	2	3.51
Civil servant	1	1.79	4	7.02
Driver	0	0	1	1.75
Office worker	4	7.14	4	7.02
Teacher	1	1.79	0	0
College student	0	0	1	1.75
Self employed	6	10.71	6	10.53
Housewife	9	16.07	11	19.30

**Table 2:** KOP scores of control and intervention groups on pre- and post-tests

	**Groups**		***T*** ** Test**		
	**Control**	**Intervention**	***t***	***df***	***P***
Pre-test	6.47 ± 2.61	6.91 ± 2.95	-0.828	111	0.409
Post-test	8.29 ± 3.25	9.48 ± 3.01	-2.017	111	0.046

**Table 3:** ANCOVA using pre-test as covariate to adjust for the post-test score

**Source of variation**	**Sum of squares**	***df***	**Mean square**	***F***	***P***
Groups	21.670	1	21.670	3.692	0.057
Error	645.631	110	5.869		
Total	10003.000	113			
